# Voxel-Based Morphometry Reveals a Correlation Between Bone Mineral Density Loss and Reduced Cortical Gray Matter Volume in Alzheimer’s Disease

**DOI:** 10.3389/fnagi.2020.00178

**Published:** 2020-06-17

**Authors:** Yumi Takano, Yasuko Tatewaki, Tatsushi Mutoh, Naoya Morota, Izumi Matsudaira, Benjamin Thyreau, Tatsuo Nagasaka, Hayato Odagiri, Shuzo Yamamoto, Hiroyuki Arai, Yasuyuki Taki

**Affiliations:** ^1^Department of Nuclear Medicine and Radiology, Institute of Development, Aging and Cancer, Tohoku University, Sendai, Japan; ^2^Department of Geriatric Medicine and Neuroimaging, Tohoku University Hospital, Sendai, Japan; ^3^Smart-Aging International Research Center, Tohoku University, Sendai, Japan; ^4^Division of Radiology, Tohoku University Hospital, Sendai, Japan; ^5^Department of Geriatrics and Gerontology, Institute of Development, Aging and Cancer, Tohoku University, Sendai, Japan

**Keywords:** MRI, osteoporosis, bone mineral density, Alzheimer’s disease, voxel-based morphometry

## Abstract

**Background**: Decreased bone mineral density (BMD) was associated with poorer cognitive function and increased risk of Alzheimer’s disease (AD). However, objective evidence for the relationship between osteoporosis and AD in humans has not been extensively described.

**Objectives**: We aimed to evaluate the relationships between BMD and the cortical volumes of brain regions vulnerable to AD; hippocampus, parahippocampal gyrus, precuneus, posterior cingulate, and angular gyrus, using voxel-based morphometry (VBM), to investigate the association between bone loss and AD.

**Methods**: A cohort of 149 consecutive elderly participants who complained of memory disturbance underwent high-resolution structural brain magnetic resonance imaging (MRI) and dual-energy X-ray absorptiometry (DXA). We used SPM12 software to conduct a voxel-based multiple regression analysis to examine the association between femoral neck BMD values and regional gray matter volume (rGMV) on structural T1-weighted MRI.

**Results**: After adjusting for subject age, gender, total brain volume (TBV), and mini-mental state examination (MMSE) scores, the multiple regression analysis showed significant correlations between BMD loss and rGMV decline in the left precuneus, which is an important neural network hub vulnerable to AD.

**Conclusion**: These data suggest that the bone and brain communicate with each other, as in “bone-brain crosstalk,” and that control of BMD factors could contribute to cognitive function and help prevent AD.

## Introduction

Both osteoporosis and Alzheimer’s disease (AD) are global problems, especially in developed countries where the proportion of the elderly population is growing. Both osteoporosis and AD increase proportionally with age and shorten life expectancy (Yoshimura et al., 2009; Compston et al., 2019).

In AD, atrophy or hypoperfusion identified on imaging modalities such as computed tomography (CT), magnetic resonance imaging (MRI), and single-photon emission tomography (SPECT) appears in specific areas of the brain. These findings are important objective biomarkers for the diagnosis of patients with AD (Ito et al., 2014) and imply that neural networks contributing to cognitive function have been disrupted.

Epidemiological studies suggest that reduced areal bone mineral density (BMD) and increased rates of bone loss are associated with cognitive decline and a higher risk of AD (Yaffe et al., 1999; Zhou et al., 2014; Kang et al., 2018; Lv et al., 2018). One explanation for this relationship is that whole-body homeostasis relies on the crosstalk between organs and that this crosstalk is essential for coordinating their activity and ensuring proper regulation of their physiological functions. Among such ideas, an interaction between bone and brain, the so-called “bone-brain crosstalk,” has emerged recently (Rousseaud et al., 2016). The bone not only regulates phosphate and calcium metabolism but also secretes an osteoblast-derived molecule (e.g., osteocalcin) that appears to be an important factor influencing the central nervous system by regulating the brain development and cognitive functions (Obri et al., 2018).

Current studies reported that low BMD is associated with lower whole-brain volume and memory deficits in early AD, suggesting that AD-related degeneration of the central nervous system may play a role in bone loss (Loskutova et al., 2009; Bae et al., 2019). In a previous study using brain SPECT perfusion images, we demonstrated hypoperfusion in the posterior cingulate cortex in elderly women suffering from both osteopenia and AD (Takano et al., 2020). Although several substantial reports have indicated relationships between osteoporosis and AD, the specific topographic features of the brain that are associated with bone loss in humans have not yet been extensively described. In particular, whether bone loss can affect regional structural alteration in AD-related regions (e.g., hippocampus, parahippocampal gyrus, temporoparietal areas, posterior cingulate gyrus, and precuneus) remains unclear. Therefore, we hypothesized that improved understanding of the associations between bone loss and topographic changes in AD-related regions would provide strategies for contributing to effective prevention and treatment of osteoporosis and AD.

Dual-energy X-ray absorptiometry (DXA) is the standard and most precise technique for BMD measurement, and BMD is the standard measure for the diagnosis of osteoporosis. In 1994, the World Health Organization (WHO) first proposed diagnostic criteria for osteoporosis, with the diagnosis of osteoporosis being based on T-score, which is the difference between the BMD value (in g/cm^2^) of an individual and the average BMD [expressed in standard deviation (SD) units] of a young adult in a reference population (Lu et al., 2016).

MRI is a useful method to visualize central nervous system alterations. In particular, advanced neuroimaging techniques, high-resolution three-dimensional (3D) structural MRI combined with spatial-normalization methods enable us to analyze the precise quantitative topological features of brain structure. Voxel-based morphometry (VBM; Ashburner et al., 2003) has been extensively used to quantitatively investigate the location and degree of structural changes in the brain associated with normal development and aging, and with pathological conditions such as AD (Matsuda, 2016). Regional gray matter volume (rGMV) can be calculated with the VBM method, which is a biomarker known to directly reflect brain function at the voxel level in the brain.

The goal of this study was to investigate the relationship between osteoporosis and AD by using VBM-based rGMV to visualize brain alterations associated with BMD.

## Materials and Methods

### Participants

This study enrolled 149 consecutive patients (age range from 65 years to 89 years) who visited the outpatient department of the Department of Geriatric Medicine and Neuroimaging at Tohoku University Hospital for a complaint of memory disturbance between February 1, 2015, and July 31, 2018. All but one male participant was right-handed. Eligible patients had brain MRI and DXA scans completed at the initial investigation. The patients’ age, body mass index (kg/m^2^), years of education, mini-mental state examination score (MMSE; Folstein et al., 1975), and Logical Memory II test score (a measure of delayed recall) from the Wechsler Memory Scale-Revised (WMS-R) were extracted from their charts (Kawano et al., 2013). The exclusion criteria for all participants were: (1) other neurological disorders; and (2) coexisting severe medical conditions or terminal diseases (e.g., stroke, Parkinson’s disease, thyroid/parathyroid disease, and cancer) that may influence the results of the imaging studies.

### Data Collection

For this single-center retrospective cohort study, all data and information regarding the patients’ clinical courses were collected and reviewed from medical records maintained by our institute.

### BMD and Skeletal Muscle Index (SMI) Measurements With DXA

BMD and skeletal muscle index (SMI) were measured from a single protocol on a whole-body DXA machine (QDR 4500a, Hologic Inc., Bedford, MA, USA; Kunitoki et al., 2019; Takano et al., 2019, 2020). DXA can provide both total and regional body composition through a three-compartment method that distinguishes total bone mineral content from soft tissue. This allows the assessment of whole-body and site-specific lean mass. SMI can then be determined from these measurements (Imboden et al., 2017). DXA images of the left femoral neck were used to calculate BMD in this study.

### Brain MRI Acquisition and Preprocessing

A 3.0-T MRI scanner (Intera Achieva 3.0T Quasar Dual, Philips) and a 32-element head coil were used to acquire a 3D T1-weighted magnetization-prepared rapid acquisition gradient echo (MPRAGE) structural image (repetition time, 8.70 ms; echo time, 3.1 ms; 8° flip angle; the field of view, 256 × 256 × 180 mm; and voxel size, 0.7 × 0.7 × 0.7 mm).

Pre-processing of the structural images was performed using Statistical Parametric Mapping software (SPM12; Wellcome Department of Cognitive Neurology, London, UK) implemented in MATLAB (Mathworks Inc., Natick, MA, USA) as described (Matsudaira et al., 2019). Using the new segmentation algorithm implemented in SPM12, the T1-weighted structural images were segmented into gray matter, white matter, and cerebrospinal fluid. The segmentations performed in this study used the default parameters, except affine regularization, which used the International Consortium for Brain Mapping (ICBM) template for the brains of European subjects. We then used the DARTEL (diffeomorphic anatomical registration through exponentiated lie algebra) registration process implemented in SPM12 to spatially normalize the six TPMs (tissue probability maps) to Montreal Neurological Institute (MNI) space, obtaining images with 1.5 × 1.5 × 1.5 mm voxels. Subsequently, all images were smoothed by convolving them with an 8-mm full width at half-maximum (FWHM) isotropic Gaussian kernel, producing the final GMV maps.

The total brain volume (TBV) was calculated by combining the gray matter images with the white matter images generated during the SPM segmentation process. Intracranial volume (ICV) was calculated by adding the segmented cerebrospinal fluid space to TBV.

### Statistical Analysis

The brain parenchymal ratio of TBV over ICV (TBV/ICV) was calculated for normalization by body size. As a partial correlation analysis, Pearson correlation coefficients with adjustment for age and MMSE scores were separately calculated to evaluate the relationship between brain parenchymal ratio and BMD.

**Figure 1:**
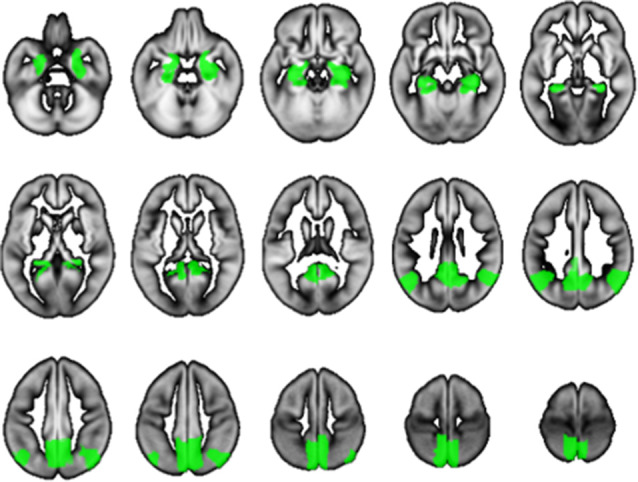
The mask used for small volume correction (SVC). The anatomical mask was created for SVC with a voxel-level significance of 0.05 corrected for multiple comparisons. Using the automated anatomical labeling atlas (Imboden et al., 2017), the single mask was composed of the hippocampus, parahippocampal gyrus, posterior cingulate cortex, precuneus, and angular gyrus, which are cortical regions associated with Alzheimer’s disease (AD).

We used SPM12 to conduct a whole-brain multiple regression analysis to examine the associations between BMD and rGMV on brain structure. Multiple regression analysis enables us to evaluate correlations between each voxel value on brain MRI and clinical data (in this case, BMD) against the whole brain. Consequently, the voxels/regions which have a statistically significant correlation with the clinical data (independent valuable) can be demonstrated on the whole brain map. The subjects’ age, gender, TBV, and MMSE score were entered as covariates of no interest in these analyses. All continuous variables were mean-centered before analysis. The initial voxel threshold was set to 0.001 uncorrected. Results were considered significant only if the voxel-level family-wise-error (FWE)-rate-corrected *P*-value was lower than 0.05, with this correcting for multiple comparisons across the whole brain using random field theory. In addition to the whole-brain analysis, anatomical masks were created for small volume correction (SVC) with a voxel-level significance of 0.05 corrected for multiple comparisons. Regions were selected for which an *a priori* hypothesis existed concerning AD: hippocampus, parahippocampal gyrus, posterior cingulate cortex, precuneus, and angular gyrus (Matsuda, 2016). All regions were defined and unified into a single mask using the automated anatomical labeling atlas (Maldjian et al., 2003) included in WFU Pick Atlas ver. 3.0.4^1^ available with SPM. This single mask was used for SVC (Figure 1).

## Results

This cross-sectional study involved 57 males and 92 females participants with an age range of 65–89 years and a mean age of 79.4 ± 5.8 (SD) years. The patients’ demographic data are presented in Table 1 according to gender. The male group showed significantly higher scores than the female group on the BMD (*P* < 0.001), SMI (*P* < 0.001), and education (*P* < 0.05), but a worse score on the MMSE (*P* < 0.05). All other demographic variables were similar between the two groups.

**Table 1:** Baseline demographic and clinical parameters.

	Total (*n* = 149)	Male (*n* = 57)	Female (*n* = 92)
Age (years)	79.6,5.6 (65–89)	79.5,5.9 (65–89)	79.6,5.4 (67–89)
BMI (kg/m^2^)	22.6, 5.9 (12.4–35.5)	19.8, 4.5 (14.7–30.6)	24.3,6.1 (12.4–35.5)
Education (years)	11.7, 2.7 (6–20)	12.3, 3.3* (6–20)	11.4,2.2 (6–16)
MMSE	22.3,4.7 (6–30)	21.4, 5.6* (6–30)	22.8,4.0 (14–30)
WMS-R (logical memory-II)	4.7,6.0 (0–22)	6.2, 7.0 (0–20)	3.9,5.4 (0–20)
BMD (g/cm^2^)	0.59,0.14 (0.29–1.00)	0.68,0.14^†^ (0.30–1.00)	0.54,0.12 (0.29–0.94)
SMI (kg/m^2^)	6.12, 1.05 (3.82–9.32)	6.75,1.08^†^ (4.02–9.32)	5.71,0.81 (3.82–8.37)

Data are expressed as mean ± SD (range) or the number of subjects. Significance male vs. female: *P < 0.05; ^†^*P* < 0.001. BMD, bone mineral density; BMI, body mass index; SD, standard deviation; MMSE, Mini-Mental State Examination; SMI, skeletal muscle mass index; WMS-R, Wechsler Memory Scale-Revised.

In the partial correlation analysis, the brain parenchymal ratio did not significantly correlate with BMD after adjusting for age or MMSE score (with age adjustment: *r* = −0.109, *P* = 0.094; with MMSE adjustment: *r* = −0.125, *P* = 0.066).

**Figure 2:**
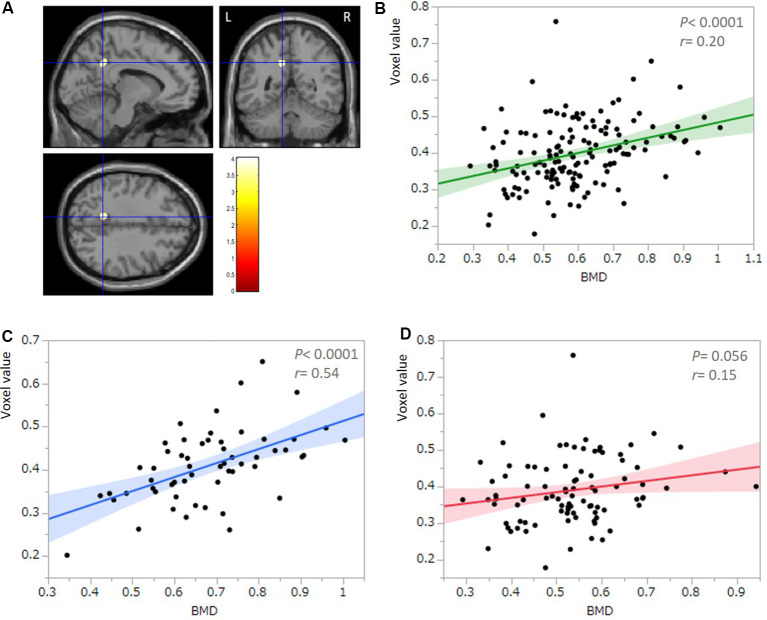
Results of multiple regression analysis with SVC.** (A)** Statistical parametrical map (thresholded at *P* < 0.001, uncorrected for display purposes) shows the results from the regression analysis of bone mineral density (BMD) against regional gray matter volume (rGMV) using voxel-based analysis. Multiple regression analysis against all participants (*n* = 149) demonstrated a significant positive correlation between BMD and rGMV in the left precuneus. The result was significant after correction for multiple comparisons using SVCs for AD-associated regions (*P* < 0.05). The color bar indicates the *t*-value. Scatter plot against all participants (*n* = 149; **B)** and the male group (*n* = 57; **C)** indicates the association between the mean voxel value of the left precuneus and BMD. **(D)** A Scatter plot against the female group (*n* = 92) indicates no significant correlation between the mean voxel value of the left precuneus and BMD.

All multiple regression analyses were conducted with adjustment for age, gender, MMSE, and TBV using SPM12. As shown in Figure 2 and Table 2, the multiple regression analysis using all participants demonstrated a significant positive correlation between BMD and rGMV in the left precuneus (MNI coordinates at peak voxel = (−11, −50, 36); *t* = 4.03, cluster size = 113, Adjusted *P* = 0.046 after SVC). Except for the left precuneus, no other regions including the right precuneus showed statistically significant correlations between BMD and rGMV with even non-corrected VBM (see Supplementary Materials). There was no significant negative association between BMD and rGMV in any region. Further multiple regression analysis on separate male and female groups demonstrated a significant positive correlation between BMD and rGMV in the left precuneus (MNI coordinates at peak voxel = (−12, −48, 38), *t* = 4.57, cluster size = 99, adjusted *P* = 0.021 after SVC) in male subjects, but no significant association between BMD and rGMV was found in any region in the female subjects (Figure 2).

**Table 2:** Voxel-based morphometry multiple regression analysis between bone mineral density and rGMV.

Structure	MNI coordinates			*t*	*P*	Cluster size
	*X*	*Y*	*Z*			
(All participants) Left precuneus	−11	−50	36	4.03	0.046	113
(Male) Left precuneus	−12	−48	38	4.17	0.021	99

*rGMV, regional gray matter volume; MNI, Montreal Neurological Institute*.

## Discussion

Our study revealed that BMD was positively correlated with rGMV in the left precuneus in elderly subjects after adjusting for covariates including age, gender, brain size, and MMSE. The left precuneus is one of the regions known to be associated with the change in AD. Also, the correlation showed gender dependance, being stronger in elderly men than in elderly women. To the best of our knowledge, this is the first report to demonstrate a correlation between reduced BMD and lower rGMV in the left precuneus in elderly subjects.

Osteoporosis and AD are multifactorial diseases that are both prevalent in elderly populations. Epidemiological studies have suggested that lower BMD measured by DXA scan is associated with cognitive decline and increased risk of AD (Yaffe et al., 2012; Rousseaud et al., 2016). Based on this relationship, the whole-body homeostasis concept of “bone-brain crosstalk” was proposed. Skeletal system-derived hormones such as osteocalcin, and brain-derived neurotrophic factor and insulin-like growth factor regulate cognitive function and effect neurogenesis (Ding et al., 2006; Obri et al., 2018). Using imaging modalities, several studies have attempted to clarify the evidence for bone-brain crosstalk in human subjects, and have mentioned the possibility that AD-related brain degeneration may affect bone remodeling or bone loss, and that AD may share common biological mechanisms including collagen type1 (Loskutova et al., 2009; Bae et al., 2019). However, little is known about the precise topological features of the brain alterations that accompany bone loss. Recently, in our work using SPECT, we demonstrated a relationship between BMD and lowered perfusion in the left posterior cingulate cortex in female subjects with osteopenia comorbid with AD (Takano et al., 2020). We hypothesized that bone loss may affect neurodegenerative functional and structural changes in the specific regions associated with AD. Such brain alteration in the AD-associated regions may be an intermediate biomarker suggesting a strong link between osteoporosis and AD. This study extends on the prior one by identifying specific regional morphological changes showing an association between BMD and AD.

Our study revealed that BMD was positively correlated with rGMV in the left precuneus, which is one of the regions vulnerable to AD pathology. The precuneus is an important hub for networks involved in cognitive functions and is involved in the default mode network (DMN). The medial prefrontal cortex, PCC, precuneus, anterior cingulate cortex, parietal cortex, and hippocampus show synchronized activation during the resting state, and together form the DMN. AD was reported to disrupt the DMN because the DMN structures are vulnerable to atrophy, deposition of amyloid and tau protein, and reduced glucose metabolism due to AD pathology (Hafkemeijer et al., 2012). This study demonstrated that BMD positively correlated with rGMV in the left precuneus, a part of the DMN, which could indicate that bone loss may have some effect on brain structure and cognitive function. However, the results of our correlation analysis suggested no direct relationship between BMD and MMSE or WMS-R, which reflect global cognitive function and episodic memory, respectively. We supposed that the link between BMD loss and AD may be associated with other implicit cognitive factors such as spatial perception, arousal, memory, and executive attention, which were not evaluated by the MMSE and WMS-R. Thus, we interpreted the link as suggesting, at least in part, a common pathological mechanism in AD, which contributes to both bone loss and decreased rGMV in the left precuneus.

Although our previous study using SPECT found that decreased rCBF in the left PCC was correlated with lower BMD, this current study using VBM showed an rGMV decrease in the left precuneus associated with lower BMD. This discrepancy in the region affected between our two studies could be explained by differences in the image resolution of the two imaging modalities. The image resolution of the SPECT was much lower than that of the MRI, with the voxel sizes being approximately 10 mm and 1–1.5 mm, respectively. In this context, the normalization procedure and regions of interests (ROIs) adopted for the SPECT study were rather coarse compared with the MRI-VBM method used in this study. The significantly associated area in this VBM study was near the posterior cingulate cortex and medial and inferior clusters within the left precuneus, which included in the left PCC-ROI mask in the previous SPECT study. We, therefore, posit that the two results from the different imaging modalities could be consistent.

Other studies have reported whole-brain volume loss in association with BMD loss (Loskutova et al., 2009; Bae et al., 2019). However, our study demonstrated no significant correlation between BMD and global brain volume after adjusting for age or MMSE score. Furthermore, Loskutova et al. (2009) reported that BMD decline was associated with low gray matter volume in several regions, including the hypothalamus, cingulate, parahippocampal gyri, left temporal gyrus, and left inferior parietal cortex, whereas our VBM analysis demonstrated that bone-loss showed a significant correlation with rGMV in the left precuneus, but not with the hypothalamus and other regions comprising the limbic system. This discrepancy could be due to several factors. First, the participants in the other work were healthy elderly subjects and those with the earliest stage of AD. In contrast, our current study included a profile of participants ranging from severe dementia to normal elderly subjects. Such differences in the participants’ backgrounds could influence the results. The other work might emphasize a very early phenomenon of bone-brain associations, whereas our study might demonstrate a more advanced stage of bone loss and its effects on the brain. Again, this current study is the first description of an association between topographical features of brain change in AD-related cortices and bone loss in an elderly subject cohort including normal subjects and those with AD. An analysis stratifying subjects according to their clinical presentations, including normal subjects, those with mild cognitive impairment, and those with dementia, is needed to clarify the effect of BMD on the brain. Second, other studies have evaluated the relationship between BMD and whole-brain volume without statistically adjusting for cognitive status. BMD has been reported to correlate with some memory scales (Loskutova et al., 2009); therefore, results showing positive correlations between BMD and whole-brain volume may be influenced by a potential confounder such as cognitive status.

Our study demonstrated that a correlation between BMD and rGMV in the left precuneus was apparent in elderly male, but not in elderly female subjects, although several studies in women suggested that low BMD was associated with a high risk of developing AD and cognitive decline (Tan et al., 2005; Yaffe et al., 2012). One possible explanation for this gender effect is the extent of the therapeutic intervention for osteoporosis. Although we did not specifically measure the treatment history and medications for osteoporosis according to the medical records, the majority of female participants in this study underwent medication therapy for osteoporosis, including vitamin D, calcium, estrogen, bisphosphonate, and molecular targeted drugs, whereas few participants in the male group underwent such therapy. BMD values in the femoral neck, such as those used in this study, are sensitive to trabecular bone remodeling and easily vary in response to therapy, being used as a biomarker for therapeutic effectiveness. Therefore, such a general improvement of BMD in response to osteoporosis treatment could have modulated our result in the female group. Indeed, our previous study using SPECT, which enrolled only female subjects with osteopenia who had not undergone treatment for osteoporosis, demonstrated a significant correlation between BMD and rCBF in the left PCC. Another possibility is that gender variations such as hormonal variations and skeletal muscle quantity could modulate the effect of bone-brain crosstalk; bone loss may have a stronger effect on structural brain alterations in elderly males than in females. Further investigations considering treatment history for osteoporosis, sex hormone values, and skeletal mass index are needed.

Our study has some limitations. First, this was a cross-sectional observational study from a single center. The possibility of selection bias cannot be excluded, and longitudinal studies are needed to confirm a causal effect between BMD loss and rGMV loss within AD-related regions. Second, the participant profile included a great variety of ages, cognitive functioning varying from severe dementia to nearly normal aging, and treatment histories for several complicating diseases. Additionally, other variables affect BMD and cognitive function, such as daily exercise and nutritional status, including vitamin D and calcium. We did not control for the effects of these other confounding variables because the sample size limited the power to adequately examine the influence of such covariates on BMD. Furthermore, our results cannot be directly applied to the normal elderly population, who are the most important strategic target for the prevention of dementia. This study should be considered a pilot study, and the external validity of the results must be confirmed by a larger prospective study with systematic and longitudinal analysis. Third, we did not evaluate the bone-related serum biomarkers such as Bone Specific Alkaline Phosphatase: bone formation marker (BAP) or Tartrate-resistant Acid Phosphatase 5b: bone resorption marker (TRACP-5b) which could directly reflect bone metabolism in this study. We presumed that such bone-related biomarkers might also attribute to brain function and morphology. We plan to investigate bone-related serum biomarkers as a role of bone-brain crosstalk in our further study.

In conclusion, the present study demonstrated that lower BMD was associated with lower rGMV in the left precuneus in an elderly population, suggesting that bone loss may influence an important hub of a neural network affected by AD. The use of imaging modalities to visualize the evidence for bone-brain crosstalk has only just begun. Given these insights, the establishment of new treatments and preventive strategies for AD by controlling for risk factors affecting osteoporosis and cognitive function could be of great clinical importance.

## Data Availability Statement

The raw data supporting the conclusions of this article will be made available by the authors, without undue reservation, to any qualified researcher.

## Ethics Approval and Registration

The studies involving human participants were reviewed and approved by the Clinical Research Ethics Committee of Tohoku University Graduate School of Medicine (2018-1-298). Written informed consent for participation was not required for this study in accordance with the national legislation and the institutional requirements. The trial was registered in the University hospital Medical Information Network Clinical Trials Registry (UMINCTR; UMIN000035762).

## Author Contributions

YTak, YTat, and TM co-designed the study. TN and HO were responsible for scanning the subjects and supported the imaging technology. YTak, YTat, NM, IM, and BT analyzed the data. YTak, YTat, and TM wrote the first draft of the manuscript. YTak, YTat, and SY were responsible for the subject’s characterization and sample collection. All authors contributed to and have approved the final manuscript.

## Conflict of Interest

The authors declare that the research was conducted in the absence of any commercial or financial relationships that could be construed as a potential conflict of interest.

## Footnotes

^1^https://www.nitrc.org/projects/wfu_pickatlas/

## Abbreviations

AD: Alzheimer’s disease
BMD: bone mineral density
CT: computed tomography
DARTEL: diffeomorphic anatomical registration through exponentiated lie algebra
DXA: dual-energy X-ray absorptiometry
ICBM: International Consortium for Brain Mapping
ICV: intracranial volume
MMSE: mini-mental state examination
MNI: Montreal Neurological Institute
MPRAGE: magnetization-prepared rapid acquisition gradient-echo
MRI: magnetic resonance imaging
rGMV: regional gray matter volume
SPECT: single-photon emission tomography
SMI: skeletal muscle index
SVC: small volume correction
SD: standard deviation
3D: three-dimensional
TBV: total brain volume
VBM: voxel-based morphometry
WMS-R: Wechsler Memory Scale-Revised
WHO: World Health Organization.
