# Supplementary material for: Voxel-Based Morphometry Reveals a Correlation Between Bone Mineral Density Loss and Reduced Cortical Gray Matter Volume in Alzheimer’s Disease
Source: Front Aging Neurosci. 2020 Jun 17;12:178. doi: 10.3389/fnagi.2020.00178 (PMC7311782; doi:10.3389/fnagi.2020.00178)
Supplement: Supplementary file 1 [file Table_1.docx]

**Supplementary Data**

We created region-of-interest (ROI) mask of the right precuneus by flip-horizontal of the existing significant voxel cluster in the left precuneus (Montreal Neurological Institute coordinates at peak voxel = [−11, −50, 36]; cluster size = 113), and measured regional grey matter volume (rGMV) within the right precuneus mask (**Fig S1**). The results of a Pearson correlation analysis showed no significant correlation between bone mineral density (BMD) and rGMV of the right precuneus (All: *r* = 0.017, *P* = 0.320; Male: *r* = 0.030, *P* = 0.826; Female: *r* = 0.057, *P* = 0.591). The results of partial correlation analysis after adjusting age, gender, total brain volume and MMSE were also no significant correlation between BMD and rGMV of the right precuneus (All: *r* = 0.028, *P* = 0.740; Male: *r* = 0.051, *P* = 0.717; Female: *r* = 0.064, *P* = 0.554).

**
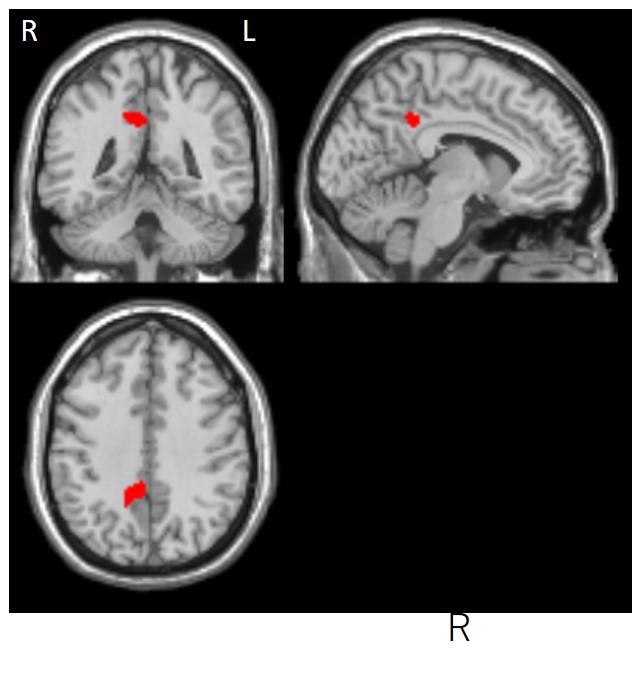
**

**figure S1.** A mask according to the ROI of the right precuneus. The right precuneus mask was created by flip-horizontal of the existing significant voxel cluster in the left precuneus with voxel-based multiple regression analysis. R, right; L, left.
